# Novel expressed sequences identified in a model of androgen independent prostate cancer

**DOI:** 10.1186/1471-2164-8-32

**Published:** 2007-01-26

**Authors:** Steven N Quayle, Heidi Hare, Allen D Delaney, Martin Hirst, Dorothy Hwang, Jacqueline E Schein, Steven JM Jones, Marco A Marra, Marianne D Sadar

**Affiliations:** 1 Genome Sciences Centre, British Columbia Cancer Agency, 600 West 10^th ^Ave., Vancouver, BC, V5Z 4E6, Canada

## Abstract

**Background:**

Prostate cancer is the most frequently diagnosed cancer in American men, and few effective treatment options are available to patients who develop hormone-refractory prostate cancer. The molecular changes that occur to allow prostate cells to proliferate in the absence of androgens are not fully understood.

**Results:**

Subtractive hybridization experiments performed with samples from an *in vivo *model of hormonal progression identified 25 expressed sequences representing novel human transcripts. Intriguingly, these 25 sequences have small open-reading frames and are not highly conserved through evolution, suggesting many of these novel expressed sequences may be derived from untranslated regions of novel transcripts or from non-coding transcripts. Examination of a large metalibrary of human Serial Analysis of Gene Expression (SAGE) tags demonstrated that only three of these novel sequences had been previously detected. RT-PCR experiments confirmed that the 6 sequences tested were expressed in specific human tissues, as well as in clinical samples of prostate cancer. Further RT-PCR experiments for five of these fragments indicated they originated from large untranslated regions of unannotated transcripts.

**Conclusion:**

This study underlines the value of using complementary techniques in the annotation of the human genome. The tissue-specific expression of 4 of the 6 clones tested indicates the expression of these novel transcripts is tightly regulated, and future work will determine the possible role(s) these novel transcripts may play in the progression of prostate cancer.

## Background

Prostate cancer is the most frequently diagnosed cancer as well as the second leading cause of cancer death among American men [1]. Androgen ablation therapy for patients with advanced prostate cancer inevitably fails as the disease progresses to an androgen-independent stage [2]. Few effective treatment options are available to these patients, and these increase survival by only a matter of months [3,4]. We examined an *in vivo *human prostate cancer tumour model to identify the underlying molecular events involved in hormonal progression. The LNCaP hollow fibre model differs from xenograft models by growing the LNCaP human prostate cancer cell line within fibres that are implanted subcutaneously in host mice [5]. These fibres prevent host cells from infiltrating, and contaminating, the tumour cell population. Upon castration of the host the LNCaP cells progress to an androgen-independent stage as determined by a rising titre of serum prostate-specific antigen (PSA), mimicking this aspect of clinical disease [5].

Many genes important in the development and progression of cancer have been identified by first detecting their altered expression at different stages of the disease. It has thus become desirable to perform high-throughput gene expression analyses to quickly assay the expression status of large numbers of genes in a given model or treatment condition. A variety of techniques are available for monitoring gene expression profiles, with microarrays and Serial Analysis of Gene Expression (SAGE) being the most widely used. However, microarray experiments are only able to monitor the expression of genes for which prior knowledge of the transcript sequence is available, and they also lack the sensitivity to detect transcripts expressed at very low levels. The SAGE technique is capable of detecting novel transcripts [6-9], but SAGE is also not optimal for detecting low abundance transcripts. In contrast, suppression subtractive hybridization includes a normalization step that enriches for rare transcripts in a population of RNAs [10,11]. Subtractive hybridization is also able to detect entirely novel transcripts for which no previous annotation exists [12,13]. Thus, subtractive hybridization is a powerful tool to detect less abundant transcripts and the novel transcripts that tend to be expressed at low levels. In support of this concept, a significant proportion of the transcripts identified by subtractive hybridization were shown to be expressed at levels below the detection limit of Affymetrix GeneChip^® ^arrays [14]. Additionally, subtractive hybridization identified a number of novel transcripts which were not represented on these arrays [14].

Gene expression changes occurring with the hormonal progression of prostate cancer have been examined in various systems (see [15-17], for example). Our goal was to utilize the LNCaP hollow fibre model to identify genes that had not been previously associated with prostate cancer. The application of subtractive hybridization resulted in the identification of a number of novel expressed sequences in this model. These sequences exhibit low protein-coding potential and low conservation across species, but RT-PCR experiments confirmed their expression in samples of prostate cancer and in a variety of human tissues.

## Results

### Novel transcripts were represented in the subtracted cDNA libraries

Suppression subtractive hybridization was used to isolate novel transcripts expressed at different stages of hormonal progression in the LNCaP hollow fibre model. This model enables the isolation and molecular analysis of prostate cells (free from contamination by host cells) at multiple stages of hormonal progression. Samples from intact mice, from mice 10 days after castration (at PSA nadir), and mice 45–60 days post-castration were used as the tester and driver samples in independent subtractive hybridization experiments. Each experiment compared different time points, and both forward and reverse subtractions were performed for each comparison.

**Figure 1:**
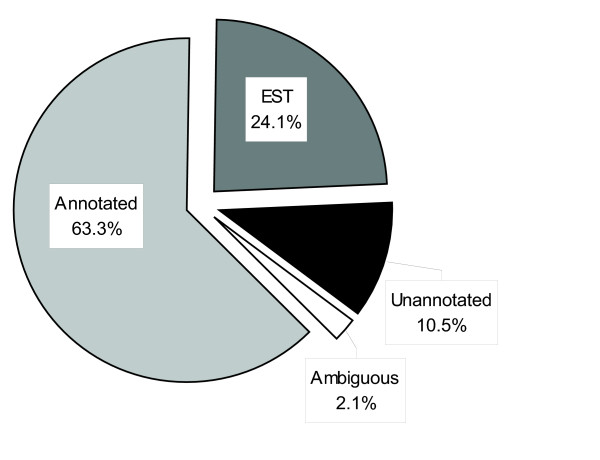
**Distribution of sequence matches in the suppression subtractive hybridization clone set**. The 237 non-redundant clones identified through sequence analysis of the subtractive hybridization libraries were mapped to the human genome (v. 35) and classified as representing an annotated Ensembl cDNA, a previously identified expressed sequence tag (EST), or an unannotated region of the genome. Clones not mapping uniquely to the genome were classified as ambiguous matches.

To identify novel genes associated with prostate cancer we examined the sequences of all 428 of the subtracted clones isolated from our subtractive hybridization experiments. These sequences were filtered to remove poor quality sequences and any clones containing less than 25 bp of sequence, more than one insert, or regions of repetitive sequence (Table 1). Of the remaining 340 clones, 103 clones contained inserts that were represented by other clones in the library. BLAST analysis of the Ensembl database (v. 35 – Nov 2005) [18] was then used to identify the transcript represented in each of the remaining 237 nonredundant clones. First, the clones were searched against the population of annotated Ensembl cDNAs, identifying 150 clones (63.3%) derived from previously annotated human cDNAs (Fig. 1). The remaining 87 clones were then mapped to the human genome using the Ensembl database, with all mappings verified using the UCSC Human Genome Browser (hg17 – May 2004) [19] and GenBank databases. This search identified 57 clones (24.1%) matching expressed sequence tags (EST) and mRNAs which had not yet been classified as Ensembl cDNAs. A clone exhibiting any overlap with a transcript was considered to be part of that transcript. Of the 30 remaining clones, 5 (2.1%) did not align uniquely to the genome, but the other 25 (10.5%) did map uniquely to unannotated regions of the human genome and were considered to represent novel transcripts (Fig. 1). These 25 clones mapped to a variety of locations, including within introns of annotated transcripts, nearby annotated transcripts, as well as intergenic regions. This analysis suggested that novel transcripts were represented in our subtractive hybridization libraries.

### Characterization of novel clone sequences

We next assessed the sequence characteristics of these novel clones to determine if there were differences between those mapping to unannotated regions of the genome relative to those clones mapping to known transcripts. Five characteristics were examined for each clone: (1) their protein coding potential, (2) their evolutionary conservation, (3) whether they showed evidence of splicing, (4) whether they demonstrated homology to known non-coding RNAs, and (5) whether they contained a poly-adenylation site. For this analysis, the 25 novel clones (Table 2) were compared to 25 clones randomly chosen from the 57 that mapped to previously identified ESTs (Table 3), and another 25 clones randomly chosen from the 150 mapping to annotated transcripts (Table 4). First, to assess the likely coding potential of each clone, the length of the longest open reading frame (ORF) was determined using the ORF Finder tool at NCBI. The clones derived from known transcripts were much more likely to contain an ORF spanning most of the sequence. It should also be noted that the sequences used for this analysis were derived from single-pass sequencing reads, and thus potential sequencing errors may interrupt longer ORFs. Next, we qualitatively assessed the degree of conservation of each of these clones using the Vertebrate and Multiz Alignment and Conservation track at UCSC [20]. This track provides a measure of evolutionary conservation of a genomic region amongst 17 vertebrate species. As predicted, clones originating from known transcripts tended to show high conservation of their respective genomic loci, while the clones representing novel transcripts mapped to regions of the genome that were poorly conserved. This was consistent with the prediction that highly conserved transcripts would be expressed by a larger number of species, thus increasing the likelihood of the transcript having been detected in previous studies.

We were also interested in whether these sequenced clones derived from spliced regions of transcripts. While over half of the annotated clones appeared to be spliced, only one clone matching an EST was spliced, and none of the novel clones showed evidence of splicing. It may be that the clones matching ESTs and unannotated regions were more frequently derived from untranslated regions of transcripts. This would be consistent with the decreased size and number of ORFs in these clones. A number of families of non-coding RNAs have also now been identified, such as ribosomal RNAs, transfer RNAs, and more recently, micro RNAs. To determine if the unannotated sequences identified here may be non-coding RNAs, these sequences were queried against the Rfam database of non-coding RNA families [21]. None of the novel sequences displayed homology to any of the known RNA families in this database. Next we examined all these clones for the presence of poly-adenylation sites. Approximately 30% of all clones, regardless of their mapping, were poly-adenylated, further supporting that the novel clones were derived from mRNA transcripts. This result also showed that the known and novel transcript fragments were equally likely to contain poly-adenylation sites, suggesting that novel clones were not specifically biased towards the 3' untranslated regions of transcripts.

### Analysis of SAGE tags in the novel clones

SAGE is a second technique that has been used to identify large numbers of novel transcripts [6,7]. We sought to determine if the 25 novel clones had been previously detected in SAGE experiments performed with human tissues. Subtracted clones matching unannotated regions of the human genome were searched for potential *Nla*III restriction sites and the 17 bp of downstream sequence was considered to represent a LongSAGE tag. Twenty of our unannotated sequences contained uniquely mapping regions which could be potential LongSAGE tags, and many clones contained multiple possible tags. We then compared our predicted LongSAGE tags to a metalibrary containing approximately 11 million uniquely mapping human SAGE tags previously identified at the B.C. Cancer Agency Genome Sciences Centre or in the Cancer Genome Anatomy Project (CGAP) at NCBI. These tags were identified in 79 SAGE libraries representing a variety of human tissues, including embryonic and haematopoietic stem cells, samples of normal and cancerous lung, brain, pancreas, breast and colon, as well as some human cancer cell lines. Only three of the 20 unannotated clones contained LongSAGE tags that were previously detected in a SAGE experiment (Table 2); none of these tags mapped to annotated transcripts. These tags, from clones 1cE10, 2dB01, and 2dD06, occurred once, thrice, and once, respectively, in the entire human LongSAGE metalibrary, providing *in silico *evidence that these clones represent novel transcripts that are expressed at very low levels.

A LongSAGE tag and a novel clone may also derive from the same transcript without the two sequences directly overlapping. We therefore looked for LongSAGE tags mapping to the 50 kb of genomic DNA sequence flanking our novel sequences to identify any neighbouring regions predicted to be expressed based on the previous SAGE experiments. The genomic neighbourhoods of all 20 of the analyzed subtracted clones contained LongSAGE tags that were not from annotated transcripts. As stated above, 3 of these clones mapped directly to a LongSAGE tag. Of the remaining 17 clones, 7 mapped within 2.5 kb of a previously detected LongSAGE tag, while the remaining 10 clones were up to 23 kb from their nearest tag. These tags also occurred at low frequency in the human metalibrary, with each tag being detected from one to three times. These regions of the genome do not appear to encode abundantly expressed transcripts, providing a possible explanation why these transcripts were not detected previously. This analysis supported the concept that our novel subtracted clones were likely to represent entirely novel transcripts.

### The novel subtracted clones are expressed by a variety of cell types

**Figure 2:**
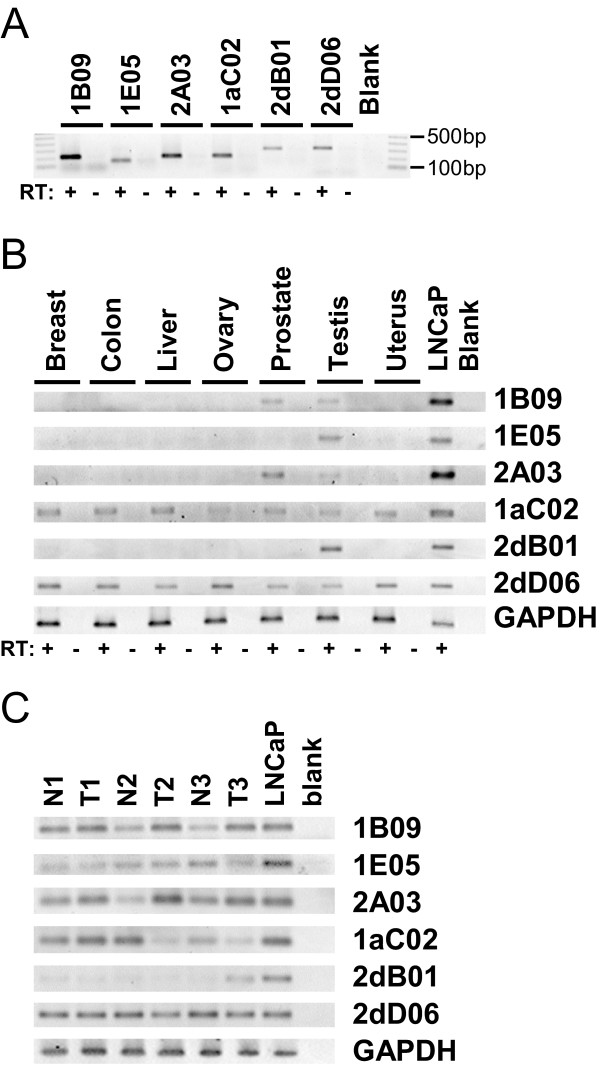
**The novel subtracted clones were expressed in a variety of human tissues**. (A) RT-PCR was performed with primer pairs specific for each of six novel clones using cDNA from the LNCaP hollow fibre model. RT indicates the presence or absence of Reverse Transcriptase. (B) RT-PCR was performed using cDNAs generated from normal human tissues with each of the six novel clones. All clones were expressed in at least one of the normal tissues. (C) RT-PCR was performed using cDNAs generated from three samples of prostate cancer (T1 – T3) and their respective matched normal samples (N1 – N3). All six of the clones were expressed in at least one of these samples.

To confirm that the novel clones detected by subtractive hybridization are naturally expressed in cells we performed a series of RT-PCR experiments. We chose 6 novel clones and examined their expression in LNCaP cells from the hollow fibre model. All six of the chosen clones were clearly expressed in LNCaP cells (Fig. 2A). The absence of bands in the reverse transcriptase (RT)-negative samples confirmed that these products did not derive from contamination with genomic DNA. The tested clones included two clones containing a previously identified LongSAGE tag, three clones for which the nearest tag was between 2 to 8 kb away, and one clone that was approximately 14 kb from the nearest LongSAGE tag. Thus, the novel transcript fragments isolated here were derived from expressed transcripts that had not previously been documented in any database.

To determine the presence or absence of these novel transcripts in various tissues we performed RT-PCR with a panel of RNAs derived from "normal" human tissues. Each of the 6 clones chosen displayed unique tissue expression profiles, with 4 clones being highly specific to the prostate and testes or the testes alone, while the remaining 2 clones were expressed in all the tissues tested (Fig. 2B). Clone 2A03 was almost exclusively expressed in the prostate, with only a faint product observed in the testes. Even the two clones observed in all the tissues tested appeared to be expressed at variable levels in each of the tissues. Thus, while the expression of some of these clones appeared higher in LNCaP cells, the expression of these novel sequences was not unique to this cell line.

Next we examined the expression of these 6 novel transcripts in three samples of prostate cancer with matched samples of "normal" prostate. While the cancer specimens were not microdissected, these samples were all scored by a pathologist to contain 65–80% tumour tissue. Five of the transcripts were expressed in both the normal and cancer specimens (Fig. 2C). Clone 2dB01 showed the most limited expression, being detectable in only one sample of cancer. The expression of this transcript is quite limited as LNCaP cells and the single cancer specimen were the only non-testicular samples in which expression could be demonstrated (Fig. 2B,C). While expression levels can not be precisely measured via RT-PCR, some of these clones appeared to show differential expression in the normal samples relative to the cancer samples. From these experiments we conclude that the novel clones derived from our subtractive hybridization experiments in fact represent previously unannotated transcripts that are expressed in a variety of human tissues.

### Five of the novel clones are part of larger untranslated regions

**Figure 3:**
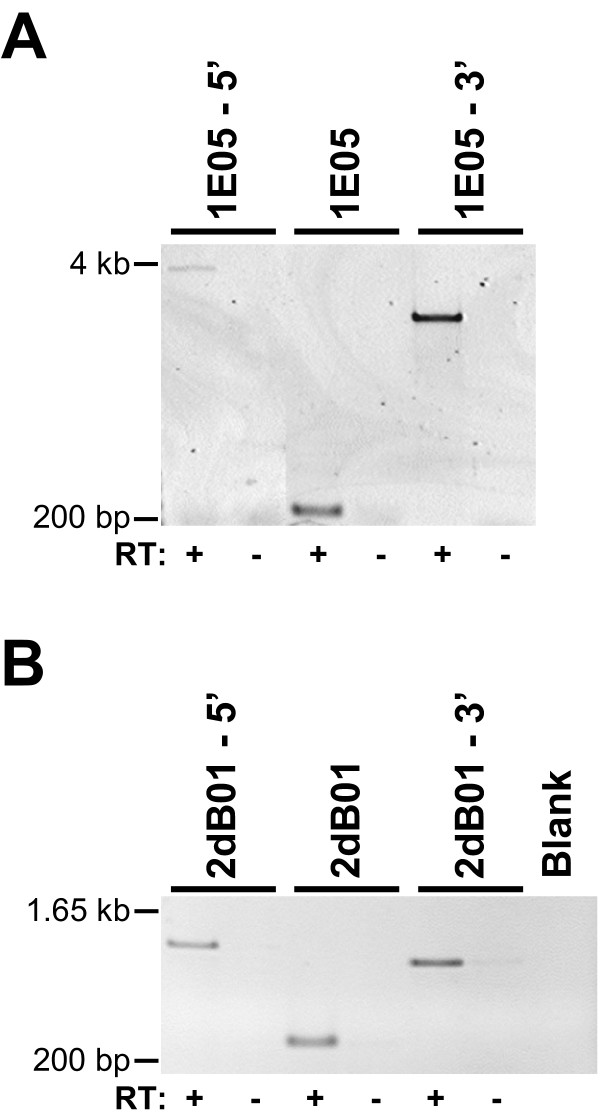
**Two of the novel clones were part of transcripts containing a neighbouring SAGE tag**. RT-PCR amplified transcript regions between clones 1E05 (A) and 2dB01 (B) and SAGE tags identified nearby each clone. RT indicates the presence or absence of Reverse Transcriptase.

The novel clones isolated here were cDNA fragments presumably derived from larger transcripts. We wanted to isolate more sequence information from the full-length transcripts to understand the possible functions of these novel transcripts. The above analysis with the human SAGE metalibrary identified a number of tags mapping near the novel clones isolated using subtractive hybridization, suggesting they may represent the same transcript. RT-PCR experiments were then performed utilizing primers spanning clones 1E05 and 2dB01 and their neighbouring LongSAGE tags. These experiments demonstrated that some of these tags were in fact derived from larger transcripts that also contained these novel subtracted clones (Fig. 3). These PCR products were not detected in reverse transcriptase negative samples, confirming they were not derived from genomic DNA. Upon sequencing of these PCR amplicons, clone 1E05 was demonstrated to be part of an additional 6 kb untranslated region (accession nos. DQ668402 and DQ668403) downstream of a previously identified, but uncharacterized, mRNA represented in the GenBank database (AL832227). Interestingly, mRNA AL832227 was originally identified in human testis tissue. This provides validation of the tissue expression profiles we observed as clone 1E05 was only detected in cDNA from testis (Fig. 2B). Finally, clone 2dB01 was part of a 1.6 kb transcript that did not contain any significant ORFs (accession no. DQ668401). This transcribed region is likely an untranslated region of a larger transcript that has yet to be identified.

Rapid Amplification of cDNA Ends (RACE) was then performed in an attempt to isolate full-length transcripts for the remaining four clones. RACE in the 3' direction successfully extended clones 1B09, 2A03, and 1aC02 (accession nos. EH613608 – EH613610) to poly-adenylation sites. Unfortunately, RACE in the 5' direction did not yield larger transcript fragments for any of these clones. In all, these experiments have isolated approximately 0.9 kb of transcript 1B09, 1.5 kb of transcript 2A03, and 2.5 kb of transcript 1aC02, indicating they too encode large non-coding regions of novel transcripts.

## Discussion

While the precise number of genes in the human genome remains unknown, it is clear that an even greater number of transcripts are produced by a myriad of alternative splicing events. The recognition of non-coding RNAs has also resulted in a greater focus on transcripts not containing open reading frames. Thus, even with the completed sequence of the genome further experiments are required to fully annotate the functional units transcribed from the genome in the different cell types during normal growth and development, as well as in diseased tissues. High-resolution tiling microarrays have been used to generate predictions of likely transcripts, many of which mapped to intergenic and intronic regions of the genome that were not previously annotated [22-24]. Similarly, approaches focused on sequencing full-length cDNAs continue to identify a large number of novel transcripts, many of which appear to be non-protein coding [25,26]. Our results here provide further evidence that a significant number of genes and transcripts remain to be identified.

Studies now suggest that the majority of the human genome is transcribed, but the function(s) of most of these transcripts has not yet been demonstrated (reviewed in [27]). The lack of defined function for these transcripts has led some to propose that they arise indirectly through spurious transcription of the genome. However, new functions of non-coding transcripts continue to be identified, indicating that these "spurious" transcripts likely have functions that have yet to be characterized. For example, steroid receptor RNA activator (SRA) was demonstrated to act as an RNA transcript to regulate the transcriptional activity of steroid nuclear receptors, including the androgen receptor [28]. Another non-coding transcript, expressed at low levels in various tissues, was recently demonstrated to regulate the nuclear trafficking of nuclear factor of T cells (NFAT), and has been renamed non-coding RNA repressor of NFAT (NRON) [29]. Such studies confirm that low-abundance, non-coding, RNA transcripts perform diverse functions and regulate multiple biological processes.

Subtractive hybridization has been used to characterize gene expression changes associated with prostate cancer [12,15,30-33], and some of these studies have also characterized novel genes that were identified from their subtracted libraries [12,13,30]. However, these reports examined only a few novel genes that were differentially expressed in their experimental systems, and few studies have used subtractive hybridization to examine changes with hormonal progression in an *in vivo *model [15]. Our study is unique in that we sequenced all the clones arising from the subtractive hybridization experiments performed with *in vivo *samples from the LNCaP hollow fibre model. This approach identified a large number of transcripts that had not previously been detected in prostate cancer cells and may be of prognostic or therapeutic value.

We identified 25 completely unannotated clones, and an additional 57 clones matching previously sequenced ESTs that were otherwise unannotated. The isolation of these ESTs specifically from prostate cancer cells may prove informative at a later date. Furthermore, we considered any overlap of an EST or annotated gene with one of our clones to signify that that clone derived from a previously identified transcript. However, several of the clones matching ESTs and annotated transcripts displayed only partial overlap with the known sequence, suggesting that the subtracted clone may still represent an unidentified splice variant of the known transcript. Most of these novel transcript fragments exhibited low sequence conservation amongst vertebrate species, though this may indicate that these transcripts are human- or primate-specific. Prostate cancer is only known to spontaneously occur in humans, rats, and some species of dogs even though the prostate organ is present in all mammals. Furthermore, the human prostate exhibits numerous morphological differences. Thus, it is likely that many transcripts required for prostate development and prostate cancer progression would not be extensively conserved throughout evolution. In support of this, the *KLK3 *gene, encoding prostate-specific antigen (PSA), is only present in primate genomes [34]; PSA is a well known clinical marker used to monitor prostate cancer progression and response to therapy. This demonstrates that evolutionary conservation alone is not predictive of potential clinical utility.

The functional relevance of these novel transcripts in the hormonal progression of prostate cancer remains to be elucidated. In our experiments, 4 of the 6 clones tested were expressed only in the normal prostate and testes; this limited tissue expression profile suggests these novel transcripts may function specifically in these organs. This also suggests the expression of these transcripts is tightly regulated, as would be expected for a functional transcript. Furthermore, a related publication from our group identified a novel variant of TMEFF2 which encodes a secreted form of the protein [35]. This alternate form of the protein was identified after a novel clone from our subtractive hybridization library (clone 2A06) was mapped to the fourth intron of the *TMEFF2 *gene. The expression of TMEFF2 has been shown to increase with progression to androgen independence [15,36], consistent with our subtractive hybridization experiments. TMEFF2 is also currently being investigated as a target for antibody-based therapy in the treatment of prostate cancer [37,38], confirming that our approach identified novel transcripts which may be of interest in the study of prostate cancer. Changes in expression of some of these transcripts may also be valuable as a marker for disease progression.

To characterize the function(s) of these novel transcripts it will first be necessary to identify the full-length cDNA sequence. Multiple techniques are available to recover full-length cDNA molecules starting from only cDNA fragments. For example, RACE is widely used to obtain full-length cDNA sequences [39]. RACE was successful in isolating further 3' sequence information for 3 of the 4 clones for which it was attempted. As in the case of clone 1E05, it is possible that all three of these clones actually derive from large 3' untranslated regions of protein coding transcripts. The large size and low expression levels of these transcripts increase the difficulty of identifying their full 5' sequence. Alternatively, we may have already identified the majority of the sequence in these novel, non-coding transcripts. Another technique to identify full-length sequence for these novel transcripts would be to screen existing cDNA libraries [6,13]. However, given the relatively low expression of these transcripts this approach would likely require extensive screening of such libraries. Two recent studies have used Northern blot analysis to detect expression of similar low-abundance, non-coding, novel transcripts [22,26]. These groups found that only 20–30% of the novel transcripts were detectable by Northern blot analysis, even when using large amounts of poly-A+ RNA, indicating that the remaining transcripts fell below the detection limit of this technique.

Margulies et al. [40] recently described a highly parallel sequencing by synthesis approach that demonstrated an increased throughput for sequencing of genomic DNA. Our group has combined sequencing by synthesis and random shotgun analysis to generate ESTs and characterize the transcriptome of LNCaP cells grown in tissue culture [41]. This study isolated approximately 180,000 ESTs, and of these, 1,900 (1.0%) mapped to the human genome in regions not previously annotated in the Ensembl database. One of these ESTs mapped directly to clone 2A03 described here, while 12 more mapped within 1.5 kb of clones 1cD03 and 2dB01. However, the remaining 22 novel clones identified here were still not detected by this alternative approach.

SAGE has also been used to identify potentially novel transcripts [6-9]. Unfortunately, while SAGE provides sufficient sequence information to accurately map the tags to the human genome, there is often little other information available to aid in the design of experiments to derive more sequence data from these novel transcripts. In contrast, subtractive hybridization provides longer sequence fragments, but it is not possible to determine the orientation of these fragments. Our data also suggest that subtractive hybridization was able to detect transcripts that had not previously been found using SAGE or high throughput sequencing by synthesis. It is possible that subtractive hybridization may be more sensitive to detecting transcripts expressed at low levels. Alternatively, subtractive hybridization may isolate those transcripts that can not be efficiently detected by SAGE, for instance transcripts lacking an NlaIII restriction site. Common results from multiple techniques gives greater confidence in the identification of novel transcripts and underlines the value of using complementary techniques to achieve a more thorough analysis of the human transcriptome.

## Conclusion

Our subtractive hybridization experiments have identified novel transcripts that are specifically expressed in the prostate and/or the urogenital tissues. It may be of clinical value to further develop these novel transcripts as prognostic or therapeutic markers for prostate cancer and hormonal progression. Additionally, characterizing such novel transcripts and transcript variants may aid in identifying and understanding the processes important in the development of androgen independent disease.

## Methods

### Cell culture

LNCaP cells obtained from Dr. L.W.K. Chung (Emory University School of Medicine, Atlanta, GA) were maintained in RPMI 1640 supplemented with 5% (v/v) fetal bovine serum (FBS) (HyClone, Logan, UT), 100 units/mL penicillin, and 100 μg/μL streptomycin. All chemicals were purchased from Sigma, unless stated otherwise.

### LNCaP hollow fibre model

The LNCaP hollow fibre model has been described in detail previously [5]. Briefly, LNCaP cells (2 × 10^7^) in RPMI 1640 with 20% (v/v) FBS were sealed inside polyvinylidine fluoride (PVDF) fibres (500 kDa molecular weight cutoff; 1 mm internal diameter; Spectrum Medical Co, Houston, TX) and incubated overnight at 37°C. The fibres were then cut into fragments of approximately 2 cm and inserted subcutaneously into anesthetized male 6–8 week old athymic nude mice (BALB/c strain) obtained from Charles River Laboratory (Montreal, Canada). Serum samples were obtained from the dorsal tail vein of mice every 7 days, and PSA levels measured by an immunoenzymatic assay (Abbott IMX, Montreal, Canada). Serum samples were always obtained prior to the performance of any procedure. After one week mice were castrated by ligation of the vas deferens through a small incision in the scrotum. Control (intact) animals were not castrated, but all other procedures were performed on the same schedule. Hollow fibres were removed on the day of castration, 10 days post-castration, and 45–60 days post-castration when serum PSA levels had risen. All fibres were immediately placed on ice, washed three times in sterile phosphate buffered saline (PBS), and wiped with sterile, moistened lab wipes. Any fibre visibly contaminated by mouse tissue was set aside. To harvest cells, 1 mL of ice-cold TRIZOL^® ^Reagent (Invitrogen, Burlington, Canada) was flushed through the fibres and the cells homogenized with a 21-G needle prior to storage at -80°C. All animal procedures were performed according to protocols approved by the Animal Care Committee at the University of British Columbia.

### Suppression subtractive hybridization

The SMART PCR cDNA Synthesis Kit (Clontech, Palo Alto, CA) was used to generate full-length cDNA from 1 μg of starting total RNA using oligo-dT primer according to the manufacturer's protocol. Suppression subtractive hybridization was then performed with the PCR-Select™ cDNA Subtraction Kit (Clontech) according to the manufacturer's protocol. Briefly, the cDNA was digested with *Rsa*I restriction endonuclease to generate fragments of approximately equal lengths. The digested cDNA was purified and split into two populations before ligation of Adaptor 1 or Adaptor 2R. An excess of driver cDNA was added to each reaction, the DNA denatured, and hybridization performed for 8 hours at 68°C. The two populations of cDNA were then combined and fresh denatured driver cDNA added before hybridizing for an additional 16 hours. The final cDNA population was subjected to two rounds of PCR to specifically amplify the differentially expressed cDNA transcripts. The efficiency of subtraction was determined by monitoring levels of the housekeeping gene glyceraldehyde-3-phosphate dehydrogenase (GAPDH) in these samples. The subtracted cDNA pools were then ligated into pCR^®^2.1-TOPO vector (Invitrogen) and transformed into competent bacteria. Positive colonies were selected and their inserts sequenced unidirectionally with T7 primer on an ABI 3700 automated sequencer. Clones containing novel inserts were further sequenced to obtain the entire insert sequence. All sequences obtained in these experiments have been deposited in dbEST [Accession numbers EC093848 – EC094057; EH613608 – EH613610] and GenBank [Accession numbers DQ668378 – DQ668403].

### RT-PCR

Total RNA was isolated from LNCaP cells maintained *in vivo *using TRIZOL^® ^Reagent according to the manufacturer's protocol. Total RNA samples from several human tissues were purchased from Stratagene (La Jolla, CA). Samples of total RNA from cases of prostate cancer, and their matched normal samples, were purchased from Genomics Collaborative (Cambridge, MA). Reverse transcription (RT) was performed using MMLV-RT (Invitrogen) with 1 μg of template RNA. Subsequent PCR reactions were performed using 1 μL of the resulting cDNA as template. The primers used to amplify the clones are summarized in Table 5. PCR products of interest were cloned into pCR^®^2.1-TOPO vector and sequenced by the NAPS facility at the University of British Columbia.

### RACE

Poly-A+ mRNA from LNCaP cells was used for RACE experiments with the Smart RACE cDNA Amplification kit (Clontech) according to the manufacturer's protocol. PCR products were gel-purified, cloned, and sequenced.

## Authors' contributions

SNQ, HH, and JES performed the subtractive hybridizations and sequenced the resulting clones. DH carried out the experiments with the LNCaP hollow fibre model and collected samples. MH completed RACE analyses, SJMJ and MAM conceived of the sequence analyses, and ADD designed and performed the analysis with the human SAGE metalibrary. All other experiments and analyses were carried out by SNQ. MDS conceived of the study, and participated in the conception, design, and interpretation of all experiments. The manuscript was written by SNQ, and JES, SJMJ, MAM, and MDS provided critical improvements. All authors read and approved the final manuscript.

## Figures and Tables

**Table 1:** Summary of sequenced cDNA clones

	Total (%)
Total sequenced clones	428
Mouse	24 (5.6)
Multiple inserts	10 (2.3)
Poor quality	54 (12.6)
Redundant	103 (24.1)
Remaining Nonredundant	237 (55.4)

**Table 2:** Sequence characteristics of unannotated clones

Clone	Accession	Length (bp)	ORF (%)*	Degree of Conservation	Spliced	polyA	Nearest SAGE Tag	Distance to SAGE (kb)	Chromosome Position	Description
11_B09	DQ668378	306	25.2	+^†^	no	no	CCAATCTTTAACTTCCT	8.3	2q31.3	100 kb from mRNA AK000023
11_E05	DQ668379	1338	8.1	+	no	no	GCATTTTATTTTTTAAG	1.9	15q11.2	4 kb from mRNA AL832227
12_A03	DQ668380	543	46.8	+	no	no	GTTCTCTGATTCCTAAG	14.5	2q31.3	50 kb from EST CF140309
12_A06	DQ668381	324	39.2	+	no	no	TGCAGGAGACTAGCAAG	11.6	2q32.3	Intron of *TMEFF2*
12_E10	DQ668383	576	46.7	+	no	no	No tag in clone	N/A	22q13.2	8 kb from mRNA BX648018
13_F09	DQ668384	454	50.7	+	no	yes	AGCCTCCTCCTCTCCTT	6.4	21q21.1	Intron of *NCAM2*
13_G01	DQ668385	261	41.0	+	no	yes	ATTTTCATTGCTTTTAT	7.2	18q22.3	Intron of *NETO1*
13_G06	DQ668386	389	32.1	+	no	yes	GAACAAAGACAGATATG	2.0	8q22.3	Intron of *GRHL2*
14_D01	DQ668387	134	77.6	+++	no	no	ATTTTATGGTTATTATT	8.7	3p14.2	Intron of *FHIT*
1a_A04	EC093934	245	25.3	+	no	no	No tag in clone	N/A	21q21.1	Intron of *NCAM2*
1a_C02	DQ668388	1853	12.4	++	no	no	TCTTACTTTTTCATTAG	2.2	5q23.1	Intron of *TNFAIP8*
1a_E09	DQ668389	796	29.5	+	no	no	TCAGATTTTGGATATCC	1.9	10p12.31	Intron of *NEBL*
1b_C12	DQ668390	555	16.8	+	no	no	GGATGGGATCAAGAGAG	17.5	14q12	Intron of *PRKD1*
1c_D03	DQ668391	292	18.5	+	no	no	No tag in clone	N/A	21q21.1	Intron of *NCAM2*
1c_E10	DQ668392	679	14.9	+	no	no	TACTTACGATTTTTCAG	0.0	8q22.1	Intron of DKFZp779L1068
1c_F02	DQ668393	475	26.7	+	no	no	TTCTAGCTCCATTGACT	1.8	8p11.22	Intron of *ADAM2*
1d_E01	DQ668394	771	26.7	+	no	no	TATACAACTGTAGATGC	0.15	16q12.1	Intron of *CDA08*
1d_F04	DQ668395	465	21.9	+++	no	yes	No tag in clone	N/A	5q21.3	Intron of *EFNA5*
2c_A06	EC094025	658	10.8	++	no	no	No tag in clone	N/A	20p12.1	Intron of *DSTN*
2c_C10	DQ668396	371	28.0	++	no	yes	TTCTAGCTCCATTGACT	6.4	8p11.22	Intron of *ADAM2*
2c_G10	DQ668397	969	19.8	+	no	no	TCAATGGAGGATGATAG	0.24	4q23	Intron of *PPP3CA*
2d_B01	DQ668398	1100	15.2	+	no	no	GAATCTGGATAAAGACT	0.0	10q21.1	87 kb from EST BG194644
2d_D06	DQ668399	598	37.0	+	no	yes	TTGTAACAAGTTAGTAA	0.0	12p13.2	Intron of mRNA K03207
2d_D09	EC094055	627	26.6	+	no	no	GCAACACTGCACTCCAG	8.8	12q21.31	Intron of *PPFIA2*
2d_E01	DQ668400	722	34.8	+	no	no	CAGAAAAAACAAAACAG	23.2	8q24.23	Intron of EST BQ226050

* Length of longest open reading frame as a percentage of the total length of each clone.

^† ^Degree of conservation of each clone: + = 0–9%; ++ = 10–29%; +++ = 30–59%; ++++ = 60–79%; and +++++ = 80–100% conservation.

**Table 3:** Sequence characteristics of clones matching previously identified ESTs

Clone	Accession	Length (bp)	ORF (%)*	Degree of Conservation	Spliced	polyA
11_C08	EC093853	470	19.6	+^†^	no	no
13_F07	EC093910	281	44.5	+	no	yes
13_G08	EC093913	126	75.4	+	no	no
14_D02	EC093923	671	27.6	+	no	no
14_G04	EC093933	521	42.4	+	no	no
1a_B02	EC093936	579	25.7	+	no	no
1a_D01	EC093941	451	20.4	++	no	no
1a_F05	EC093946	478	19.9	+++++	no	yes
1a_H09	EC093956	683	33.7	+	no	no
1b_A09	EC093959	619	30.4	++	no	no
1b_A11	EC093961	284	63.0	+	no	yes
1b_C01	EC093963	584	27.9	+	no	no
1c_A02	EC093968	514	25.5	++	no	yes
1c_D12	EC093979	215	59.5	+++++	no	no
1c_E05	EC093980	429	49.4	+	yes	yes
1c_G06	EC093989	525	26.1	+	no	no
1d_C12	EC094007	596	22.0	++	no	yes
1d_G02	EC094018	351	36.5	+++++	no	no
2c_B04	EC094028	484	21.5	+	no	yes
2c_B10	EC094032	427	44.0	+	no	no
2c_D10	EC094037	487	37.0	+	no	no
2c_F04	EC094043	384	45.8	+	no	yes
2c_H03	EC094044	642	48.0	+	no	no
2d_B10	EC094049	299	54.8	+++	no	no
2d_D07	EC094054	299	60.5	+	no	no

* Length of longest open reading frame as a percentage of the total length of each clone.

^† ^Degree of conservation of each clone: + = 0–9%; ++ = 10–29%; +++ = 30–59%; ++++ = 60–79%; and +++++ = 80–100% conservation.

**Table 4:** Sequence characteristics of clones matching Ensembl cDNAs

Clone	Accession	Length (bp)	ORF (%)*	Degree of Conservation	Spliced	polyA
11_F09	EC093861	149	100	+^†^	no	yes
11_G07	EC093866	169	86.4	++++	yes	no
11_G09	EC093868	323	43.3	++	no	yes
12_F01	EC093889	426	91.3	++	yes	no
12_F10	EC093893	276	74.6	++++	yes	no
12_G09	EC093898	279	49.1	++	no	no
12_G11	EC093899	433	73.2	+++++	yes	yes
14_C11	EC093921	269	94.1	++++	yes	no
14_D07	EC093926	77	96.1	+++++	no	no
14_E12	EC093929	318	43.1	++++	no	yes
14_F09	EC093932	180	100	+++	yes	no
1a_B07	EC093937	477	69.0	+++++	yes	yes
1a_E11	EC093945	655	33.7	++++	yes	no
1c_B12	EC093972	555	32.3	+++	yes	yes
1c_D05	EC093975	325	27.4	+++	no	yes
1c_D07	EC093977	425	37.9	++	no	no
1d_B08	EC094000	408	34.3	++++	no	no
1d_D04	EC094008	245	100	+++++	no	no
1d_E07	EC094012	555	89.2	+++++	yes	no
1d_E11	EC094014	670	60.6	++	no	yes
1d_F08	EC094016	297	91.9	+++++	yes	no
1d_H01	EC094020	701	44.8	+++++	yes	no
2c_B03	EC094027	92	100	+++++	yes	no
2c_D08	EC094036	475	37.7	+++++	no	no
2d_B03	EC094046	388	29.9	+	no	no

* Length of longest open reading frame as a percentage of the total length of each clone.

^† ^Degree of conservation of each clone: + = 0–9%; ++ = 10–29%; +++ = 30–59%; ++++ = 60–79%; and +++++ = 80–100% conservation.

**Table 5:** Primers used for RT-PCR

Clone		Sequence (5'-3')
1B09	F	CACAGGAGACCCTGTCTTACCT
	R	AAGCTCTTGCTAGGCATGTAGG
1E05	F	AATAGATTGGCAGGCCTTTG
	R	TGGGATGAGCAGGATATCAA
2A03	F	AGAGATGCAAACGGACGAAC
	R	TCACTTACTGGCTCCTGCAC
1AC02	F	AAGGTTTCCATTGCATCAGG
	R	CCTGAAAGGCTGGTCTTCAA
2DB01	F	CCACAACTTGGAAGCAATCA
	R	TTCCCTGTCCCCTAACTCCT
2DD06	F	TGGCCATCATCAAGTCGATA
	R	GAGGGATGGTGAAATCACTG
*GAPDH*	F	CCGAGCCACATCGCTCAGA
	R	CCCAGCCTTCTCCATGGTG

## References

[B1] Jemal A, Murray T, Ward E, Samuels A, Tiwari RC, Ghafoor A, Feuer EJ, Thun MJ (2005). Cancer statistics, 2005. CA Cancer J Clin.

[B2] Pirtskhalaishvili G, Hrebinko RL, Nelson JB (2001). The treatment of prostate cancer: an overview of current options. Cancer Pract.

[B3] Tannock IF, de Wit R, Berry WR, Horti J, Pluzanska A, Chi KN, Oudard S, Theodore C, James ND, Turesson I, Rosenthal MA, Eisenberger MA (2004). Docetaxel plus prednisone or mitoxantrone plus prednisone for advanced prostate cancer. N Engl J Med.

[B4] Petrylak DP, Tangen CM, Hussain MH, Lara PN, Jones JA, Taplin ME, Burch PA, Berry D, Moinpour C, Kohli M, Benson MC, Small EJ, Raghavan D, Crawford ED (2004). Docetaxel and estramustine compared with mitoxantrone and prednisone for advanced refractory prostate cancer. N Engl J Med.

[B5] Sadar MD, Akopian VA, Beraldi E (2002). Characterization of a New in Vivo Hollow Fiber Model for the Study of Progression of Prostate Cancer to Androgen Independence. Mol Cancer Ther.

[B6] Velculescu VE, Zhang L, Vogelstein B, Kinzler KW (1995). Serial analysis of gene expression. Science.

[B7] Saha S, Sparks AB, Rago C, Akmaev V, Wang CJ, Vogelstein B, Kinzler KW, Velculescu VE (2002). Using the transcriptome to annotate the genome. Nat Biotechnol.

[B8] Chen J, Sun M, Lee S, Zhou G, Rowley JD, Wang SM (2002). Identifying novel transcripts and novel genes in the human genome by using novel SAGE tags. Proc Natl Acad Sci U S A.

[B9] Wahl MB, Heinzmann U, Imai K (2005). LongSAGE analysis significantly improves genome annotation: identifications of novel genes and alternative transcripts in the mouse. Bioinformatics.

[B10] Diatchenko L, Lau YF, Campbell AP, Chenchik A, Moqadam F, Huang B, Lukyanov S, Lukyanov K, Gurskaya N, Sverdlov ED, Siebert PD (1996). Suppression subtractive hybridization: a method for generating differentially regulated or tissue-specific cDNA probes and libraries. Proc Natl Acad Sci U S A.

[B11] Gurskaya NG, Diatchenko L, Chenchik A, Siebert PD, Khaspekov GL, Lukyanov KA, Vagner LL, Ermolaeva OD, Lukyanov SA, Sverdlov ED (1996). Equalizing cDNA subtraction based on selective suppression of polymerase chain reaction: cloning of Jurkat cell transcripts induced by phytohemaglutinin and phorbol 12-myristate 13-acetate. Anal Biochem.

[B12] Xu J, Stolk JA, Zhang X, Silva SJ, Houghton RL, Matsumura M, Vedvick TS, Leslie KB, Badaro R, Reed SG (2000). Identification of differentially expressed genes in human prostate cancer using subtraction and microarray. Cancer Res.

[B13] Xu J, Kalos M, Stolk JA, Zasloff EJ, Zhang X, Houghton RL, Filho AM, Nolasco M, Badaro R, Reed SG (2001). Identification and characterization of prostein, a novel prostate- specific protein. Cancer Res.

[B14] Cao W, Epstein C, Liu H, DeLoughery C, Ge N, Lin J, Diao R, Cao H, Long F, Zhang X, Chen Y, Wright PS, Busch S, Wenck M, Wong K, Saltzman AG, Tang Z, Liu L, Zilberstein A (2004). Comparing gene discovery from Affymetrix GeneChip microarrays and Clontech PCR-select cDNA subtraction: a case study. BMC Genomics.

[B15] Mohler JL, Morris TL, Ford OH, Alvey RF, Sakamoto C, Gregory CW (2002). Identification of differentially expressed genes associated with androgen-independent growth of prostate cancer. Prostate.

[B16] Chen CD, Welsbie DS, Tran C, Baek SH, Chen R, Vessella R, Rosenfeld MG, Sawyers CL (2004). Molecular determinants of resistance to antiandrogen therapy. Nat Med.

[B17] Holzbeierlein J, Lal P, LaTulippe E, Smith A, Satagopan J, Zhang L, Ryan C, Smith S, Scher H, Scardino P, Reuter V, Gerald WL (2004). Gene expression analysis of human prostate carcinoma during hormonal therapy identifies androgen-responsive genes and mechanisms of therapy resistance. Am J Pathol.

[B18] Hubbard T, Andrews D, Caccamo M, Cameron G, Chen Y, Clamp M, Clarke L, Coates G, Cox T, Cunningham F, Curwen V, Cutts T, Down T, Durbin R, Fernandez-Suarez XM, Gilbert J, Hammond M, Herrero J, Hotz H, Howe K, Iyer V, Jekosch K, Kahari A, Kasprzyk A, Keefe D, Keenan S, Kokocinsci F, London D, Longden I, McVicker G, Melsopp C, Meidl P, Potter S, Proctor G, Rae M, Rios D, Schuster M, Searle S, Severin J, Slater G, Smedley D, Smith J, Spooner W, Stabenau A, Stalker J, Storey R, Trevanion S, Ureta-Vidal A, Vogel J, White S, Woodwark C, Birney E (2005). Ensembl 2005. Nucleic Acids Res.

[B19] Kent WJ, Sugnet CW, Furey TS, Roskin KM, Pringle TH, Zahler AM, Haussler D (2002). The human genome browser at UCSC. Genome Res.

[B20] Siepel A, Bejerano G, Pedersen JS, Hinrichs AS, Hou M, Rosenbloom K, Clawson H, Spieth J, Hillier LW, Richards S, Weinstock GM, Wilson RK, Gibbs RA, Kent WJ, Miller W, Haussler D (2005). Evolutionarily conserved elements in vertebrate, insect, worm, and yeast genomes. Genome Res.

[B21] Griffiths-Jones S, Moxon S, Marshall M, Khanna A, Eddy SR, Bateman A (2005). Rfam: annotating non-coding RNAs in complete genomes. Nucleic Acids Res.

[B22] Kampa D, Cheng J, Kapranov P, Yamanaka M, Brubaker S, Cawley S, Drenkow J, Piccolboni A, Bekiranov S, Helt G, Tammana H, Gingeras TR (2004). Novel RNAs identified from an in-depth analysis of the transcriptome of human chromosomes 21 and 22. Genome Res.

[B23] Bertone P, Stolc V, Royce TE, Rozowsky JS, Urban AE, Zhu X, Rinn JL, Tongprasit W, Samanta M, Weissman S, Gerstein M, Snyder M (2004). Global identification of human transcribed sequences with genome tiling arrays. Science.

[B24] Cheng J, Kapranov P, Drenkow J, Dike S, Brubaker S, Patel S, Long J, Stern D, Tammana H, Helt G, Sementchenko V, Piccolboni A, Bekiranov S, Bailey DK, Ganesh M, Ghosh S, Bell I, Gerhard DS, Gingeras TR (2005). Transcriptional maps of 10 human chromosomes at 5-nucleotide resolution. Science.

[B25] Imanishi T, Itoh T, Suzuki Y, O'Donovan C, Fukuchi S, Koyanagi KO, Barrero RA, Tamura T, Yamaguchi-Kabata Y, Tanino M, Yura K, Miyazaki S, Ikeo K, Homma K, Kasprzyk A, Nishikawa T, Hirakawa M, Thierry-Mieg J, Thierry-Mieg D, Ashurst J, Jia L, Nakao M, Thomas MA, Mulder N, Karavidopoulou Y, Jin L, Kim S, Yasuda T, Lenhard B, Eveno E, Suzuki Y, Yamasaki C, Takeda J, Gough C, Hilton P, Fujii Y, Sakai H, Tanaka S, Amid C, Bellgard M, Bonaldo Mde F, Bono H, Bromberg SK, Brookes AJ, Bruford E, Carninci P, Chelala C, Couillault C, de Souza SJ, Debily MA, Devignes MD, Dubchak I, Endo T, Estreicher A, Eyras E, Fukami-Kobayashi K, Gopinath GR, Graudens E, Hahn Y, Han M, Han ZG, Hanada K, Hanaoka H, Harada E, Hashimoto K, Hinz U, Hirai M, Hishiki T, Hopkinson I, Imbeaud S, Inoko H, Kanapin A, Kaneko Y, Kasukawa T, Kelso J, Kersey P, Kikuno R, Kimura K, Korn B, Kuryshev V, Makalowska I, Makino T, Mano S, Mariage-Samson R, Mashima J, Matsuda H, Mewes HW, Minoshima S, Nagai K, Nagasaki H, Nagata N, Nigam R, Ogasawara O, Ohara O, Ohtsubo M, Okada N, Okido T, Oota S, Ota M, Ota T, Otsuki T, Piatier-Tonneau D, Poustka A, Ren SX, Saitou N, Sakai K, Sakamoto S, Sakate R, Schupp I, Servant F, Sherry S, Shiba R, Shimizu N, Shimoyama M, Simpson AJ, Soares B, Steward C, Suwa M, Suzuki M, Takahashi A, Tamiya G, Tanaka H, Taylor T, Terwilliger JD, Unneberg P, Veeramachaneni V, Watanabe S, Wilming L, Yasuda N, Yoo HS, Stodolsky M, Makalowski W, Go M, Nakai K, Takagi T, Kanehisa M, Sakaki Y, Quackenbush J, Okazaki Y, Hayashizaki Y, Hide W, Chakraborty R, Nishikawa K, Sugawara H, Tateno Y, Chen Z, Oishi M, Tonellato P, Apweiler R, Okubo K, Wagner L, Wiemann S, Strausberg RL, Isogai T, Auffray C, Nomura N, Gojobori T, Sugano S (2004). Integrative annotation of 21,037 human genes validated by full-length cDNA clones. PLoS Biol.

[B26] Dalla E, Mignone F, Verardo R, Marchionni L, Marzinotto S, Lazarevic D, Reid JF, Marzio R, Klaric E, Licastro D, Marcuzzi G, Gambetta R, Pierotti MA, Pesole G, Schneider C (2005). Discovery of 342 putative new genes from the analysis of 5'-end-sequenced full-length-enriched cDNA human transcripts. Genomics.

[B27] Willingham AT, Gingeras TR (2006). TUF love for "junk" DNA. Cell.

[B28] Lanz RB, McKenna NJ, Onate SA, Albrecht U, Wong J, Tsai SY, Tsai MJ, O'Malley BW (1999). A steroid receptor coactivator, SRA, functions as an RNA and is present in an SRC-1 complex. Cell.

[B29] Willingham AT, Orth AP, Batalov S, Peters EC, Wen BG, Aza-Blanc P, Hogenesch JB, Schultz PG (2005). A strategy for probing the function of noncoding RNAs finds a repressor of NFAT. Science.

[B30] Hubert RS, Vivanco I, Chen E, Rastegar S, Leong K, Mitchell SC, Madraswala R, Zhou Y, Kuo J, Raitano AB, Jakobovits A, Saffran DC, Afar DE (1999). STEAP: a prostate-specific cell-surface antigen highly expressed in human prostate tumors. Proc Natl Acad Sci U S A.

[B31] Stubbs AP, Abel PD, Golding M, Bhangal G, Wang Q, Waxman J, Stamp GW, Lalani EN (1999). Differentially expressed genes in hormone refractory prostate cancer: association with chromosomal regions involved with genetic aberrations. Am J Pathol.

[B32] Porkka KP, Visakorpi T (2001). Detection of differentially expressed genes in prostate cancer by combining suppression subtractive hybridization and cDNA library array. J Pathol.

[B33] Chetcuti A, Margan SH, Russell P, Mann S, Millar DS, Clark SJ, Rogers J, Handelsman DJ, Dong Q (2001). Loss of annexin II heavy and light chains in prostate cancer and its precursors. Cancer Res.

[B34] Lundwall A, Clauss A, Olsson AY (2006). Evolution of kallikrein-related peptidases in mammals and identification of a genetic locus encoding potential regulatory inhibitors. Biol Chem.

[B35] Quayle SN, Sadar MD (2006). A truncated isoform of TMEFF2 encodes a secreted protein in prostate cancer cells. Genomics.

[B36] Glynne-Jones E, Harper ME, Seery LT, James R, Anglin I, Morgan HE, Taylor KM, Gee JM, Nicholson RI (2001). TENB2, a proteoglycan identified in prostate cancer that is associated with disease progression and androgen independence. Int J Cancer.

[B37] Afar DE, Bhaskar V, Ibsen E, Breinberg D, Henshall SM, Kench JG, Drobnjak M, Powers R, Wong M, Evangelista F, O'Hara C, Powers D, DuBridge RB, Caras I, Winter R, Anderson T, Solvason N, Stricker PD, Cordon-Cardo C, Scher HI, Grygiel JJ, Sutherland RL, Murray R, Ramakrishnan V, Law DA (2004). Preclinical validation of anti-TMEFF2-auristatin E-conjugated antibodies in the treatment of prostate cancer. Mol Cancer Ther.

[B38] Zhao XY, Schneider D, Biroc SL, Parry R, Alicke B, Toy P, Xuan JA, Sakamoto C, Wada K, Schulze M, Muller-Tiemann B, Parry G, Dinter H (2005). Targeting tomoregulin for radioimmunotherapy of prostate cancer. Cancer Res.

[B39] Frohman MA, Dush MK, Martin GR (1988). Rapid production of full-length cDNAs from rare transcripts: amplification using a single gene-specific oligonucleotide primer. Proc Natl Acad Sci U S A.

[B40] Margulies M, Egholm M, Altman WE, Attiya S, Bader JS, Bemben LA, Berka J, Braverman MS, Chen YJ, Chen Z, Dewell SB, Du L, Fierro JM, Gomes XV, Godwin BC, He W, Helgesen S, Ho CH, Irzyk GP, Jando SC, Alenquer ML, Jarvie TP, Jirage KB, Kim JB, Knight JR, Lanza JR, Leamon JH, Lefkowitz SM, Lei M, Li J, Lohman KL, Lu H, Makhijani VB, McDade KE, McKenna MP, Myers EW, Nickerson E, Nobile JR, Plant R, Puc BP, Ronan MT, Roth GT, Sarkis GJ, Simons JF, Simpson JW, Srinivasan M, Tartaro KR, Tomasz A, Vogt KA, Volkmer GA, Wang SH, Wang Y, Weiner MP, Yu P, Begley RF, Rothberg JM (2005). Genome sequencing in microfabricated high-density picolitre reactors. Nature.

[B41] Bainbridge MN, Warren RL, Hirst M, Romanuik T, Zeng T, Go A, Delaney A, Griffith M, Hickenbotham M, Magrini V, Mardis ER, Sadar MD, Siddiqui AS, Marra MA, Jones SJ (2006). Analysis of the prostate cancer cell line LNCaP transcriptome using a sequencing-by-synthesis approach. BMC Genomics.

